# Social class and metabolic syndrome in populations from Tunisia and Spain

**DOI:** 10.1186/s13098-015-0084-6

**Published:** 2015-10-13

**Authors:** Fadoua Gannar, Antonio Cabrera de León, Buenaventura Brito Díaz, María Del Cristo Rodríguez Pérez, Itahisa Marcelino Rodríguez, Fatma Ben Dahmen, Mohsen Sakly, Nabil Attia

**Affiliations:** Research Unit ‘Integrated Physiology’, Laboratory of Biochemistry-Human Nutrition, Faculty of Sciences of Bizerte, UR11ES33, Carthage University, Tunis, Tunisia; Research Unit, Nuestra Señora de la Candelaria University Hospital, University of La Laguna, Santa Cruz de Tenerife, Spain; Internal Medicine Department, Mohamed Taher Maamouri Hospital, Nabeul, Tunisia

**Keywords:** Metabolic syndrome, Social class, Tunisian population, Islanders

## Abstract

**Background:**

There is an increasing prevalence of obesity and metabolic syndrome (MS) in developing countries. It has been shown the relationship between social class and MS in developed countries. The objective of our study was to compare the association of social class with the prevalence of MS in a developing country (Tunisia, region of Cap-Bon) and a developed one (Spain, Canary Islands).

**Methods:**

Cross-sectional study of 6729 Canarian and 393 Tunisian individuals. Social class was measured with the income, crowding and education (ICE) model, which includes family income, household crowding and education level. Logistic regression models adjusted by age estimated the risk by odds ratio (OR) and confidence interval (CI 95 %) of MS according to social class.

**Results:**

MS prevalence was higher in Tunisian (50 %) than in Canarian women (29 %; p = 0.002), with no significant differences between men. For Canarian women, being in the highest social class was a protective factor against MS (OR = 0.39; CI 95 % 0.29–0.53) and all its components. The Canarian population and the Tunisian women, showed a significant linear trend (p < 0.001) of MS to decrease when social class increased.

**Conclusion:**

High social class is a protective factor from MS and its components within the Canarian population and the Tunisian women. Our results suggest that the socioeconomic transition in a developing country like Tunisia can improve the population health in a sex-specific manner.

## Background

The metabolic syndrome (MS) is a cluster of metabolic abnormalities that include central obesity, high blood pressure, dyslipidemia and increased fasting plasma glucose [1] that represent risk factors for type 2 diabetes, cardiovascular disease (CVD) and atherosclerosis [2]. Increasing prevalence of obesity is assumed to contribute to the rise of MS, thus becoming a global epidemic [3].

Social class is a strong determinant of inequalities in health [4]. Socio-economic status have been linked with prevalence of MS in different populations [5]. In developing countries, like Tunisia, a high proportion of people are facing some new problems as hypertension, obesity, diabetes and tobacco smoking [6]. Few studies in Tunisia have investigated the implication of socio-economic status in the prevalence of MS [7, 8]. On the other side, in developed countries the high prevalence of obesity and MS became an epidemic a couple of decades ago [9], and the close relationship between social class and MS has been corroborated [10]. In Spain, the Canary Islands have a high prevalence of obesity and MS [11, 12], and the incidence of diabetes is 1.5-fold higher than in continental Spain [13]; in this region a decreasing trend of MS when social class increases has been described, with the social being measured by the income, crowding and education (ICE) model [14].

The aim of this study was to examine whether social class is associated with MS in a different manner when comparing two populations, Tunisian and Canarian, which have a different level of development.

## Methods

### Study design

The participants in this cross-sectional study were from data collected in two independent surveys: the “CDC cohort of the Canary Island” (Cancer-Diabetes-Cardiovascular disease in these Islands) and participants from the province of Cap-Bon-northeastern Tunisia. The CDC cohort is a random sample of the adult general population (18–75 years). The total sample of the CDC study (n = 6.729) was recruited between January 2000 and December 2005. Participants were chosen randomly from the public health census in the seven Canary Islands, which covers 99 % of the population [11].

The Tunisian participants were residents in the province of Cap-Bon (northeastern-Tunisia), whose reference hospital was the Regional Hospital of Nabeul. They were 393 adults randomly recruited from the general population census, and also aged between 18 and 75 years. All individuals were sent a letter informing them about the aims of the research and inviting them to participate. They were given an appointment to visit the Department of Internal Medicine at the Hospital of Nabeul, and they were enrolled between December 2013 and May 2014. All objectives and procedures were explained to participants and an informed consent was obtained. We used the same protocol and questionnaire that had been applied for the Canarian population. The study was approved by the Bioethics Committee of Nuestra Señora de La Candelaria University Hospital and the regional Hospital of Nabeul-Tunisia.

### Measures

Briefly, the CDC Questionnaire was used to record data on personal and family antecedents of health problems and lifestyle factors. Details of the cohort are available at http://www.cdcdecanarias.org. In both cohorts, after informed consent had been obtained, participants were weighted (kg) and measured (cm) in light clothing after removing their footwear. Waist circumference was measured at a point halfway between the lowest rib and the iliac crest with the subjects breathing normally. Participants were stratified by gender and age into three strata (18–34, 35–64, and 65–75 years). Blood pressure was also recorded and fasting samples of venous blood were drawn.

### Biochemical determinations

All samples were centrifuged in situ at 2000 rpm for 10 min at room temperature and were transported daily to the hospital laboratory. Glycemia and lipids [total cholesterol, high-density lipoprotein cholesterol (HDLc) and triglycerides] were measured within 24 h of the blood draw using enzymatic methods. The results were recorded as serum concentrations in mg/dL.

### Metabolic syndrome

According to the international consensus [1], participants were considered with MS if they had three or more of the following five components: (1) abdominal obesity (waist circumference ≥80 cm in women and ≥94 cm in men); (2) elevated triglycerides ≥150 mg/dL (or drug treatment for elevated triglycerides); (3) reduced HDLc <40 mg/dL for men or <50 mg/dL for women; (4) hypertension: systolic pressure ≥130 mmHg or diastolic pressure ≥85 mmHg (or antihypertensive drug treatment); (5) hyperglycemia ≥100 mg/dL (or drug treatment for elevated glucose).

### Social class assessment

Information on education level, family income, and crowding index was collected. Social class was analyzed according to the ICE (income, crowding and education) model, obtained from the equation: (family income in quintiles + [3 × level of education] + [2 × household crowding index]). This model has been validated and produces a continuous quantitative variable that indicates higher social class with higher value [14].

### Statistical analyzes

The results are reported for the continuous variables as the mean ± standard deviation and, for the categorized variables, as percentages (%) plus 95 % confidence intervals. Chi squared tests were used to compare proportions among groups and linear trend across social class quintiles. For each category, we considered it had a statistical significance if *p* value was less than 0.05. To determine whether there was an association between quintiles of ICE model and the prevalence of MS, and with each component of MS separately, we stratified by ethnicity and sex and used logistic regression models adjusted by age; thus we obtained odds ratio (OR) with its intervals and *p* value. All statistical analyses were performed with SPSS software (version 21, in Spanish).

## Results

The MS and social class were measured in 7122 participants (Tunisian = 393 and Canarian = 6729). MS prevalence was higher in Tunisian than in Canarian women (50 versus 29 %, p = 0.002), with no significant differences between men. Stratifying by country, (Table 1) shows the comparison between gender prevalences of the variables analyzed: In the Canary Islands, MS was more prevalent in men than women (32 versus 29 % in women, p = 0.011), but within the Tunisian population the prevalence was higher in women (50 versus 39 % in men; p = 0.031). Hypertension was more frequent in men in both populations (p ≤ 0.001). The distribution of social class significantly differed in Canarian men and women, but this difference was not significant in the Tunisian population.

**Table 1:** Distribution (%) of the studied variables by gender in both populations

	Tunisian (n = 393)			Canarian (n = 6729)		
	Men (n = 174)	Women (n = 219)	*P*	Men (n = 2913)	Women (n = 3816)	*P*
MS	38.5	50.0	0.031	31.8	29.0	0.011
Abdominal obesity	47.1	92.7	<0.001	53.2	65.3	<0.001
Hyperglycemia	6.9	6.8	0.985	6.3	3.0	<0.001
Hypertriglyceridemia	32.7	32.4	0.943	32.1	18.5	<0.001
Low HDL-c	76.7	82.5	0.227	28.8	38.1	<0.001
Hypertension	59.6	42.7	0.001	58.5	44.2	<0.001
Age ≤34 years	17.2	17.4	0.003	28.7	29.4	0.816
Age 35–64 years	51.1	65.3		68.3	67.6	
Age ≥65 years	31.6	17.4		3.1	3.0	
ICE1	26.1	25.2	0.606	25.4	20.3	<0.001
ICE2	18.1	17.2		22.9	26.1	
ICE3	20.3	17.8		15.7	18.6	
ICE4	21.7	19.0		20.5	19.2	
ICE5	13.8	20.9		15.5	15.8	

*MS* metabolic syndrome, *HDL*-*c* high density lipoprotein cholesterol, *ICE1, ICE2, ICE3, ICE4, ICE5* quintiles of social class

When we stratified the population by country (Table 2), we noted that in Tunisia the MS prevalence was significantly higher in women only in quintiles 1 and 2 of social class. Instead of this, in the Canarian population the MS prevalence was lower in women only in quintiles 4 and 5. MS showed a significant linear t end to decrease when social class increased, only in women (p < 0.001).

**Table 2:** Distribution (%) of the MS by gender, according to the quintiles of social class in the Tunisian and Canarian populations

		Tunisian (n = 393)			Canarian (n = 6729)		
		Men	Women	P	Men	Women	P
ICE1	MS	30.3	62.9	0.007	35.9	39.7	0.138
ICE2	MS	30.4	60.9	0.038	37.7	34.7	0.232
ICE3	MS	45.5	42.3	0.827	32.0	31.7	0.905
ICE4	MS	40.0	37.0	0.826	27.3	19.7	0.001
ICE5	MS	35.3	32.1	0.828	21.9	13.2	<0.001

*MS* metabolic syndrome, *ICE1, ICE2, ICE3, ICE4, ICE5* quintiles of social class

**Fig. 1:**
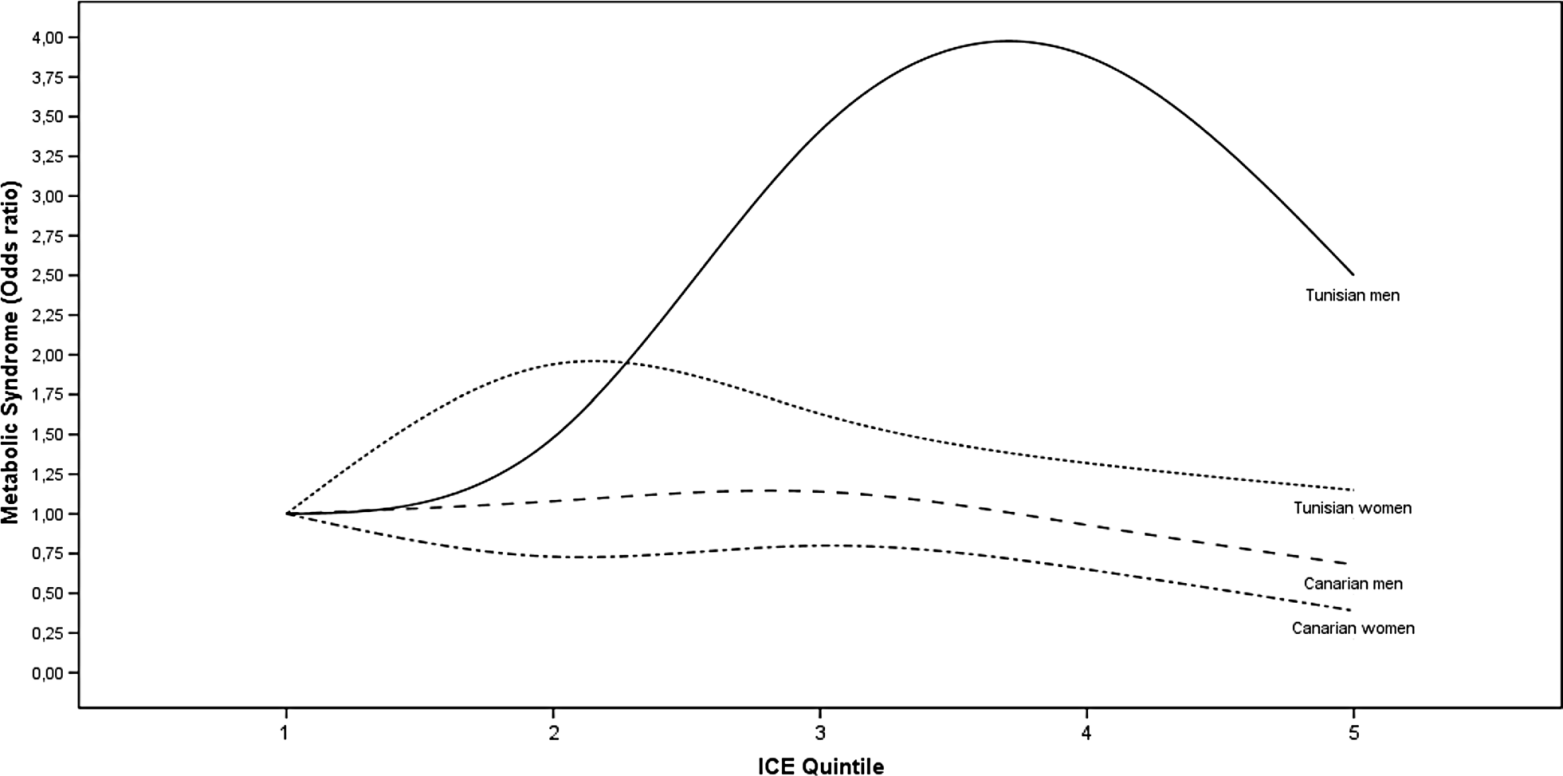
Age adjusted risk (OR) of MS in each quintile of social class for women and men from Cap Bon-region of Tunisia and Canary Islands-Spain

Tables 3, 4, 5 and 6 presents the results of logistic regression models adjusting by age the relationship of social class with the MS, after stratifying gender and country. Ageing was a risk marker of MS in every stratus. This inverse association of MS with social class was particularly strong in the Canarian women (OR = 0.39 for ICE5), and most of the MS criteria had also a lower risk in their highest social class. In the Canarian men the association between MS and social class was not so strong (OR = 0.67 for ICE5), and only two criteria (abdominal obesity and hyperglycemia) were also significantly protected in the ICE5 level. All together, the models showed that only in the Canarian population there was a significant lower risk of MS in the ICE5. However, in Tunisian women there was also a significant trend to decrease the MS risk from ICE2 to ICE5 (p < 0.001). Figure 1 shows the trends of the age-adjusted MS risk according to social class quintiles for men and women of both populations.

**Table 3:** Logistic regression model to adjust by age the relationship of social class with MS and its criteria in the Tunisian men

	Dependent variables					
	MS	Abdominal obesity	Hyperglycemia	Low HDL-c	Hypertrigliceridemia	Hypertension
Age (years)	1.05 (1.02–1.08)	1.04 (1.01–1.06)	1.04 (0.99–1.09)	0.99 (0.95–1.02)	1.02 (0.99–1.04)	1.04 (1.01–1.06)
ICE1	1	1	1	1	1	1
ICE2	1.48 (0.44–5.01)	1.15 (0.38–3.48)	0.89 (0.14–5.51)	0.89 (0.17–4.54)	1.17 (0.34–4.01)	1.66 (0.53–5.17)
ICE3	3.41 (0.99–11.72)	3.81 (1.27–11.43)	0.43 (0.04–4.30)	0.66 (0.12–3.50)	1.95 (0.61–6.27)	1.62 (0.54–4.87)
ICE4	3.88 (1.05–14.27)	2.39 (0.77–7.38)	2.31 (0.43–12.39)	0.38 (0.07–1.91)	1.91 (0.57–6.42)	1.58 (0.51–4.90)
ICE5	2.50 (0.64–9.81)	2.94 (0.87–9.91)	0.00 (0)	1.11 (0.16–7.77)	3.71 (1.05–13.12)	1.13 (0.34–3.71)

*MS* metabolic syndrome, *HDL*-*c* high density lipoprotein cholesterol, *ICE1, ICE2, ICE3, ICE4, ICE5* quintiles of social class

**Table 4:** Logistic regression model to adjust by age the relationship of social class with the MS and its criteria in the Tunisian women

	Dependent variables					
	MS	Abdominal obesity	Hyperglycemia	Low HDL-c	Hypertrigliceridemia	Hypertension
Age (years)	1.08 (1.04–1.11)	1.06 (1.01–1.11)	1.05 (0.98–1.11)	1.02 (0.98–1.06)	1.02 (0.99–1.05)	1.08 (1.04–1.11)
ICE1	1	1	1	1	1	1
ICE2	1.94 (0.56–6.74)	2.29 (0.18–28.68)	1.11 (0.17–7.28)	1.07 (0.21–5.40)	1.75 (0.63–4.90)	1.25 (0.40–3.90)
ICE3	1.63 (0.45–5.85)	1.05 (0.15–7.57)	2.58 (0.35–19.09)	1.87 (0.35–9.95)	1.25 (0.40–3.85)	0.39 (0.11–1.38)
ICE4	1.32 (0.36–4.77)	1.38 (0.18–10.42)	0.00 (0)	0.57 (0.14–2.38)	1.04 (0.33–3.30)	0.93 (0.27–1.18)
ICE5	1.15 (0.31–4.26)	1.06 (0.16–7.17)	1.52 (0.17–13.60)	1.21 (0.26–5.56)	0.66 (0.20–2.20)	0.47 (0.13–1.64)

*MS* metabolic syndrome, *HDL*-*c* high density lipoprotein cholesterol, *ICE1, ICE2, ICE3, ICE4, ICE5* quintiles of social class

**Table 5:** Logistic regression models to adjust by age the relationship of social class with the MS and its criteria in the Canarian men

	Dependent variables					
	MS	Abdominal obesity	Hyperglycemia	Low HDL-c	Hypertrigliceridemia	Hypertension
Age (years)	1.05 (1.04–1.06)	1.06 (1.05–1.07)	1.06 (1.05–1.07)	1.00 (0.99–1.01)	1.03 (1.02–1.03)	1.06 (1.05–1.06)
ICE1	1	1	1	1	1	1
ICE2	1.08 (0.86–1.37)	0.78 (0.62–0.99)	0.75 (0.50–1.13)	1.00 (0.79–1.27)	1.01 (0.81–1.27)	1.11 (0.88–1.41)
ICE3	1.14 (0.87–1.49)	0.91 (0.70–1.18)	1.04 (0.65–1.66)	1.05 (0.81–1.37)	1.07 (0.82–1.38)	0.99 (0.76–1.28)
ICE4	0.93 (0.72–1.20)	0.80 (0.63–1.02)	0.87 (0.55–1.37)	0.91 (0.71–1.17)	0.87 (0.68–1.12)	1.01 (0.80–1.29)
ICE5	0.67 (0.51–0.90)	0.60 (0.47–0.78)	0.21 (0.09–0.50)	0.92 (0.70–1.21)	0.80 (0.61–1.04)	1.10 (0.85–1.42)

*MS* metabolic syndrome, *HDL*-*c* high density lipoprotein cholesterol, *ICE1, ICE2, ICE3, ICE4, ICE5* quintiles of social class

**Table 6:** Logistic regression models to adjust by age the relationship of social class with the MS and its criteria in the Canarian women

	Dependent variables					
	MS	Abdominal obesity	Hyperglycemia	Low HDL-c	Hypertrigliceridemia	Hypertension
Age (years)	1.09 (1.08–1.09)	1.07 (1.07–1.08)	1.07 (1.05–1.09)	1.01 (1.00–1.02)	1.04 (1.04–1.05)	1.09 (1.09–1.10)
ICE1	1	1	1	1	1	1
ICE2	0.72 (0.58–0.90)	0.88 (0.69–1.13)	0.78 (0.47–1.28)	0.80 (0.66–0.98)	0.84 (0.66–1.06)	0.84 (0.67–1.04)
ICE3	0.80 (0.63–1.02)	0.89 (0.69–1.15)	0.56 (0.30–1.05)	0.72 (0.58–0.89)	0.92 (0.71–1.20)	1.08 (0.85–1.37)
ICE4	0.65 (0.50–0.85)	0.55 (0.43–0.71)	0.79 (0.41–1.52)	0.74 (0.59–0.92)	0.76 (0.57–1.01)	0.77 (0.60–0.99)
ICE5	0.39 (0.29–0.53)	0.30 (0.23–0.39)	0.49 (0.21–1.13)	0.45 (0.35–0.57)	0.55 (0.40–0.77)	0.50 (0.38–0.65)

*MS* metabolic syndrome, *HDL*-*c* high density lipoprotein cholesterol, *ICE1, ICE2, ICE3, ICE4, ICE5* quintiles of social class

## Discussion

The prevalence of MS was higher in the Tunisian than in the Canarian population. This shows a very rapid spread of the western pattern of chronic diseases towards developing countries. The prevalence of MS is very high in Tunisian women in the lowest social class and very low in Canarian women in the highest social class. Excepting the Tunisian men, there is a clear trend of MS frequency an MS risk to decrease when social class increases.

The MS is considered a problem of worldwide proportions, affecting both developed and underdeveloped countries, in a rapidly progressive way [15]. The present study’s prevalence of MS in Tunisia was higher than those reported in other populations in Oman (21 %) [16], and Iran (33.1 %) [17]. The high prevalence of the MS in Tunisian women has been reported in previous studies [18], and it is significantly higher than in Tunisian males; this difference is mainly attributable to the very high frequency of abdominal obesity in Tunisian women. However, in the Canarian population MS was significantly more frequent in males, which is attributable to their higher prevalence of hyperglycemia, hypertriglyceridemia, and hypertension. In both populations, MS was more frequent in middle-aged adults (34–64) compared to other groups as previously described [19].

Abdominal obesity was significantly more frequent in women than in men in both populations, with particularly high prevalences in Tunisian and Canarian women (93 and 65 %, respectively). This large proportion of women exceeding the waist circumference limit, may be explained by their great number of pregnancies in Tunisia, and their general lack of participation in physical activities [20, 21]. In fact, women also showed a very high frequency of low HDLc (83 % in Tunisian women) that can be related with a sedentary lifestyle. Meanwhile, men in both countries were similar regarding abdominal obesity, which was present in about half the men and can be considered as the main nucleus of MS.

One of the principal findings of the present study is the trend of MS prevalence, and MS age-adjusted risk, to decrease when social class increases. The significant impact of social class on disease makes its definition and measurement of critical importance [22]. The ICE model has been validated to measure social class [14] and it detected in Tunisia a very high prevalence of MS in women in the lowest social classes, producing a significant difference between both genders (only in ICE1 and ICE2). Meanwhile, in the Canary Islands the very low prevalence of MS in women in the highest social classes produced also a significant distance between genders (only in ICE4 and ICE5). This common trend can be related to the epidemiologic transition, which is achieved chiefly by socioeconomic improvements (as in non-developed countries) or by public health programs (as has been the case of currently developing countries). To the best of our knowledge this is the first comparative study of social class and MS between regions of Spain and Tunisia. Certainly, both countries are distant on a data-based international scale such as the Global Gender Gap Index (respectively 29th and 123th out of 142 countries [23]. Nevertheless Tunisia is known to be one of the most progressive Arab countries regarding gender legislation and empowerment of women [24], and this can reduce the distance in the context of the large-scale trends of globalization, modernization and societal changes that countries are currently experiencing [25].

As mentioned in the literature [26, 27], our results are in line with previous studies of socioeconomic factors in Tunisia. However, the interactions between the epidemiologic and socioeconomic transition are complex [28]; the lack of significant trend for MS through social class quintiles in Tunisian men can be related to this complexity of socioeconomic transition, which has different rates for each sex to change its lifestyle. Furthermore, ethnicity and genetic profile, eating habits, culture and lifestyle influence the prevalence of MS and its components too [29]. In addition, women with high social class are generally more aware of the importance of health and fitness, and so trend to consume a healthy diet and practice sports. However, men with high social class lead a more sedentary lifestyle and are more likely to smoke and drink alcohol and consume rich foods [7]. Anyway, the greatest risks of developing MS were more visible in the Tunisian population and especially in men. Why the socioeconomic protection is still not present in Tunisian men deserves further studies. It may be that among high social class Tunisian men it is not yet widely spread the feeling that fattening difficult the enjoyment of life and for one’s capacity to produce in work, family and social relations. All across the world women take more care about health than men. In fact, the protection of MS we have found in the Canarian high social classes, was more important in Canarian women than men. Using the ICE model, a high social class is today more protective against MS in the Canarian population, but there is also a significant trend in the Tunisian women.

Our study has some limitations. The population sample we could recruit in Tunisia was smaller than the Canarian sample. When stratifying for ethnicity or gender, we lost some statistical power. Another limitation is that participants were from the province of Cap-Bon-northeastern Tunisia, which is not representative of the Tunisian population. However, the Tunisian sample size has been big enough to detect some important differences and trends across social classes. The ICE model had been validated only in Spanish populations before. This model has the advantage of include income, crowding, and education level, and excluding labor occupations, and it permits a more easy international comparison. However we acknowledge that we did not performed a previous validation in Tunisia, which can be a limitation also.

We conclude that in the Canarian population and Tunisian women the prevalence and risk of MS decreases when social class increases. Our results suggest that the socioeconomic transition in a developing country like Tunisia can improve the population health in a sex-specific manner.

## Abbreviations

MS: metabolic syndrome
CVD: cardiovascular disease
NCD: non-communicable diseases
ICE index: Income, Crowding and Education index
CDC cohort: cancer-diabetes-cardiovascular disease in the Canary Islands
JIS: joint interim statement
WC: waist circumference
TG: triglycerides
HDL-c: high density lipoprotein-cholesterol
HTA: hypertension
ICE1, ICE2, ICE3, ICE4, ICE5: quintiles of ICE index
